# Complementary Roles of Artificial Intelligence and Disease-Specific Optical Diagnosis During Ulcerative Colitis Surveillance: A Prospective Tandem Colonoscopy Study

**DOI:** 10.3390/jcm15187290

**Published:** 2026-09-19

**Authors:** Andrea Cassinotti, Valentina Zadro, Matteo Ferraris, Marco Parravicini, Thomas P. Chapman, Stefano La Rosa, Sergio Segato

**Affiliations:** 1 Gastroenterology and Digestive Endoscopy Unit, Ospedale di Circolo e Fondazione Macchi, ASST Sette Laghi, 21100 Varese, Italy; valezadro@gmail.com (V.Z.); matteoferraris97@gmail.com (M.F.); marco.parravicini@asst-settelaghi.it (M.P.); sergio.segato@asst-settelaghi.it (S.S.); 2 Department of Gastroenterology, St Richard’s and Worthing Hospitals, University Hospitals Sussex NHS Foundation Trust, West Sussex PO19 6SE, UK; tomchap@doctors.org.uk; 3 Pathology Unit, Department of Medicine and Technological Innovation, University of Insubria, 21100 Varese, Italy; stefano.larosa@asst-settelaghi.it; 4 Pathology Unit, Ospedale di Circolo e Fondazione Macchi, ASST Sette Laghi, 21100 Varese, Italy

**Keywords:** artificial intelligence, ulcerative colitis, Kudo-IBD, optical diagnosis, surveillance

## Abstract

**Objectives:** Linked-colour imaging/blue-laser imaging (LCI/BLI) and CAD-EYE artificial intelligence have been developed for sporadic colorectal neoplasia, but their role in ulcerative colitis (UC) surveillance remains uncertain. We evaluated CAD-EYE-assisted detection and optical characterization using conventional and disease-specific classifications in UC. **Methods:** In this prospective tandem study, patients with UC undergoing surveillance colonoscopy were examined sequentially using white-light imaging (WLI), LCI, and LCI with CAD-EYE. Lesions were characterized using BLI with Kudo, NICE, and Kudo-IBD classifications, followed by CAD-EYE characterization. Detection and characterization performance were assessed using miss rates and diagnostic accuracy. **Results:** Among 82 patients, 281 lesions, including 22 neoplastic lesions, were identified. The lesion miss rate decreased from 5.7% with WLI to 2.5% with LCI, while no lesions were missed during the CAD-EYE-assisted withdrawal. However, the fixed examination sequence and absence of prospectively recorded withdrawal times precluded isolation of CAD-EYE’s incremental contribution. The neoplasia miss rate was numerically lower with CAD-EYE than with WLI and LCI, although not significantly. Kudo-IBD showed the highest diagnostic performance, with sensitivity, specificity, and positive and negative predictive values of 90.9%, 85.3%, 34.5%, and 99.1%, respectively. Accuracy was significantly higher with KUDO-IBD than with all other methods (all *p* < 0.001). **Conclusions:** In this sequential protocol, CAD-EYE-assisted imaging was associated with complete observed lesion detection, whereas Kudo-IBD provided superior optical characterization. These findings support complementary AI-assisted detection and disease-specific optical assessment. Larger studies with independent examination sequences and prospectively recorded withdrawal times, together with external validation of Kudo-IBD, are needed.

## 1. Introduction

Patients with ulcerative colitis (UC) are at increased risk of colorectal cancer (CRC) and undergo regular surveillance colonoscopy to allow early recognition and treatment of neoplastic lesions, thus reducing CRC-related morbidity and mortality [1]. Nevertheless, surveillance colonoscopy in UC remains considerably more challenging than conventional colorectal cancer screening. Chronic inflammation, post-inflammatory mucosal remodelling, regenerative epithelial changes, and inflammatory lesions frequently obscure subtle neoplastic lesions or mimic dysplasia, thereby reducing both lesion detection and diagnostic confidence.

Dye-based chromoendoscopy (DCE) has traditionally been regarded as the reference standard for dysplasia surveillance because of its ability to enhance subtle mucosal abnormalities [2]. However, its routine implementation has been limited by increased procedure time, technical complexity, additional costs, and heterogeneous adoption across endoscopy units. Consequently, high-definition (HD) equipment and virtual chromoendoscopy (VCE) techniques, including NBI, FICE and i-SCAN, have emerged as practical alternatives that enhance mucosal contrast without requiring dye application [3,4] and are now incorporated into recent international recommendations for IBD surveillance [1,3].

While lesion detection represents the first essential step of surveillance, accurate real-time characterization is an equally important challenge [3,4], because it directly influences biopsy strategy, endoscopic resection, and subsequent patient management. Conventional optical classifications, including the Kudo pit pattern and the Narrow-band Imaging International Colorectal Endoscopic (NICE) classification, were originally developed for sporadic colorectal neoplasia [5,6]. However, their diagnostic performance may decrease in the context of inflamed mucosa for UC, in which chronic inflammatory changes, regenerative alterations, and post-inflammatory scarring frequently modify the mucosal and vascular patterns [4]. Disease-specific classifications, such as the Kudo-IBD classification, were developed to address these limitations by incorporating endoscopic features characteristic of inflammatory bowel disease, with the aim of improving discrimination between inflammatory and neoplastic lesions [4,7,8,9].

Artificial intelligence (AI) has recently become one of the most important technological advances in gastrointestinal endoscopy. Computer-aided detection (CADe) systems consistently improve adenoma detection during CRC screening by identifying subtle mucosal abnormalities that may escape visual recognition, while computer-aided diagnosis (CADx) systems provide real-time optical prediction of lesion histology. Among currently available platforms, CAD-EYE (Fujifilm, Tokyo, Japan) has demonstrated good performance for both lesion detection and optical characterization in the general population [10,11]. However, evidence regarding its use during surveillance colonoscopy in patients with UC remains limited. Importantly, currently available CAD-EYE algorithms have not been specifically trained using UC-associated dysplasia, and their performance may therefore be influenced by the complex inflammatory background characteristic of chronic colitis. Moreover, the CAD-EYE AI system is equipped with two VCE technologies, i.e., linked-colour imaging (LCI) and blue-laser imaging (BLI), which have been validated for CRC screening in the general population, but not yet fully assessed in IBD [12,13].

To date, most studies have evaluated advanced imaging, artificial intelligence, or optical classifications separately. Little research has explored their integration within a structured surveillance strategy, and the respective contributions of AI-assisted detection and disease-specific optical diagnosis remain poorly defined in UC surveillance.

Therefore, the present study aimed to evaluate the complementary role of sequential advanced imaging modalities, AI-assisted lesion detection, and disease-specific optical diagnosis during surveillance colonoscopy in patients with UC. Specifically, we compared the lesion detection performance of WLI, LCI and CAD-EYE-assisted colonoscopy, and assessed the diagnostic accuracy of CAD-EYE, the Kudo pit pattern, the NICE classification, and the Kudo-IBD classification for real-time optical characterization using histology as the reference standard. We hypothesized that artificial intelligence and disease-specific optical diagnosis provide complementary rather than competing contributions.

## 2. Materials and Methods

### 2.1. Study Design

This prospective, single-centre study enrolled consecutive patients with UC undergoing surveillance colonoscopy at the Gastroenterology and Digestive Endoscopy Unit of Varese University Hospital, Italy. The study was approved by the local Ethics Committee and all patients provided informed consent.

The study was designed as a prospective tandem colonoscopy diagnostic accuracy study, in which multiple imaging modalities were sequentially evaluated during the same examination, using histology as the reference standard. Reporting follows the recommendations of the Standards for Reporting Diagnostic Accuracy Studies (STARD 2015) whenever applicable [14].

**Figure 1:**
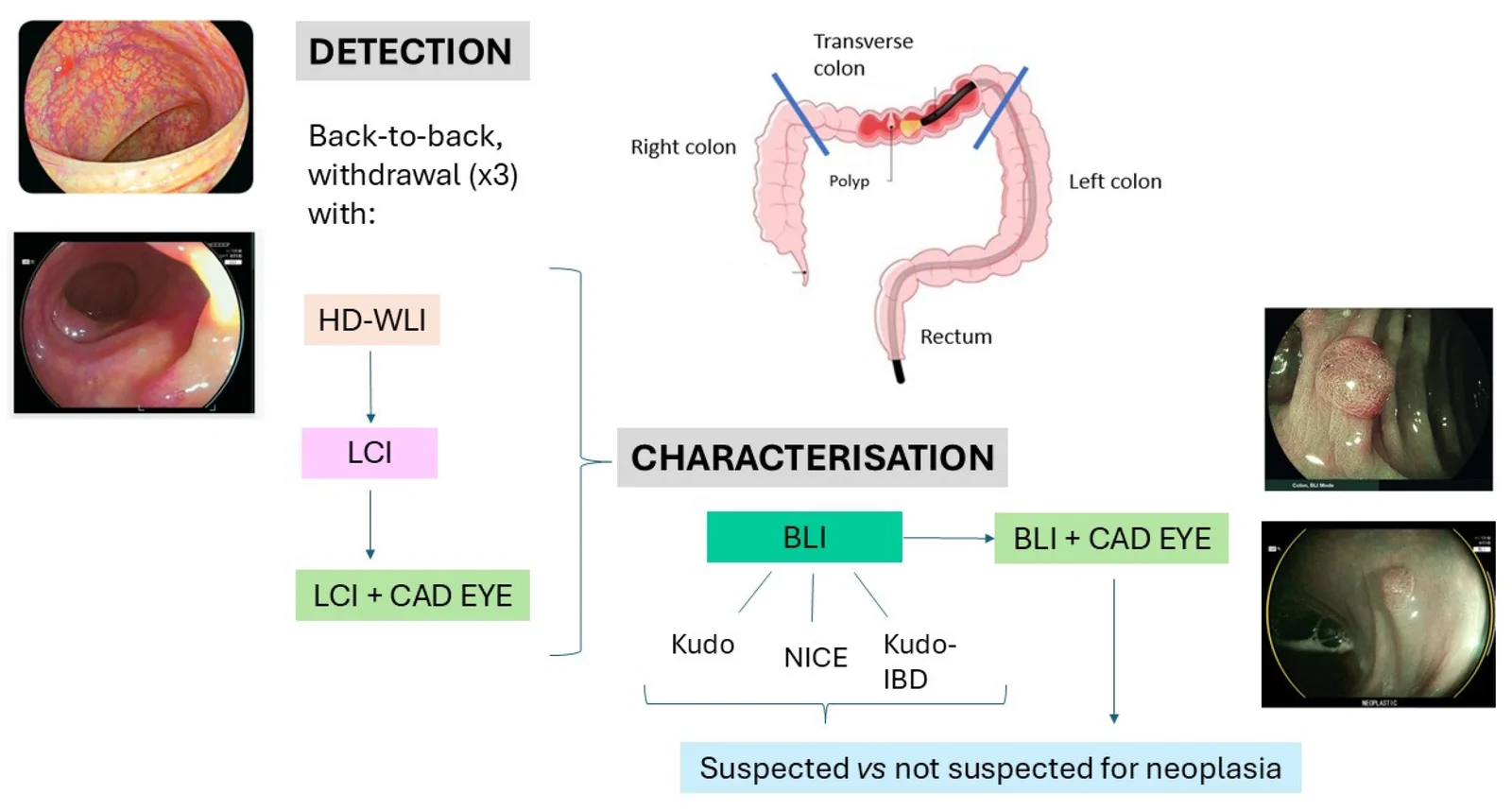
Study design. WLI = white-light imaging. LCI = linked-colour imaging. BLI = blue-light imaging.

Three technologies for lesion detection (HD-WLI vs. LCI vs. LCI+CAD-EYE) and four endoscopic classifications (Kudo, NICE, Kudo-IBD, CAD-EYE) for lesion characterization with BLI were compared in vivo (Figure 1).

The primary endpoint was lesion and neoplasia detection performance, assessed by lesion and neoplasia miss rate during sequential withdrawal using WLI, LCI, and CAD-EYE. Secondary endpoints included the diagnostic performance of optical characterization using CAD-EYE, the Kudo pit pattern classification, the NICE classification, and the disease-specific Kudo-IBD classification.

### 2.2. Inclusion and Exclusion Criteria

Inclusion criteria were patients aged 18–80 years, a diagnosis of UC according to international guidelines, and disease duration ≥8 years from onset of symptoms. For the lesion characterization substudy, only patients with at least one visible lesion were included. Any severity of clinical or endoscopic activity was permitted to reflect real-world practice. Exclusion criteria were non-correctable coagulopathy, hereditary polyposis syndromes, primary sclerosing cholangitis and poor bowel preparation (Boston score < 2 in any segment).

### 2.3. Endoscopic Protocol

All examinations were performed using the Fujifilm ELUXEO™ 7000 endoscopy platform equipped with the CAD-EYE^®^ artificial intelligence system (Fujifilm, Tokyo, Japan). Examinations were performed by three trained endoscopists (AC, VZ, MP). Two had more than 15 years of experience in IBD and surveillance colonoscopy, while the third had 5 years of dedicated experience in IBD endoscopy. Before study initiation, the endoscopists underwent protocol calibration. The Kudo-IBD classification was developed by one of the investigators (AC), who trained the other endoscopists in its application.

Following caecal intubation, a predefined sequential withdrawal protocol was performed for every patient, as described in previous pragmatic studies [15,16]. The first withdrawal used HD-WLI, the second consisted of WLI insertion followed by LCI withdrawal. During the first two withdrawal phases, the CAD-EYE system remained deactivated. A third withdrawal was then performed with CAD-EYE activated in computer-aided detection (CADe) mode. During this phase, the AI algorithm continuously analyzed the endoscopic video stream and autonomously generated real-time visual alerts whenever a suspicious lesion was identified.

Once detected, each lesion was evaluated in BLI mode and classified as suspected or not suspected for neoplasia according to NICE, Kudo and Kudo-IBD. CAD-EYE characterization was finally recorded.

Lesions identified during each withdrawal phase were recorded prospectively in real time in an electronic database, including anatomical location, estimated size, and endoscopic morphology. During subsequent withdrawal phases, the endoscopy team assessed whether observed lesions corresponded to lesions previously identified, based on the recorded characteristics and comparison with stored endoscopic images when necessary. Lesion matching was therefore performed during the procedure and was not based on independent blinded adjudication. All three withdrawal phases were completed before biopsy or endoscopic resection. After completion of the optical assessment, lesions were characterized and sampled or resected according to the final clinical assessment and routine clinical practice.

Withdrawal was slow and uniform. Dye spraying was not used.

### 2.4. Endoscopic Classifications

For each lesion, the following endoscopic variables were prospectively recorded in a dedicated electronic database: anatomical location, lesion size, Paris morphology, local Mayo Endoscopic Score (MES), Kudo-IBD modifiers (vascular pattern, pits heterogeneity, fibrin cap), optical classifications (Kudo, NICE, Kudo-IBD) and lesion visibility.

Lesion visibility was graded using a predefined 4-point visibility score which evaluates vessels, margins and surface pattern, ranging from 0 to 4 (0 = missed lesion; 1 = barely visible; 2 = visible with difficulty; 3 = clearly visible; 4 = optimally visible) [17]. For detection analyses, lesions assigned a visibility score of 0 were considered missed lesions.

**Figure 2:**
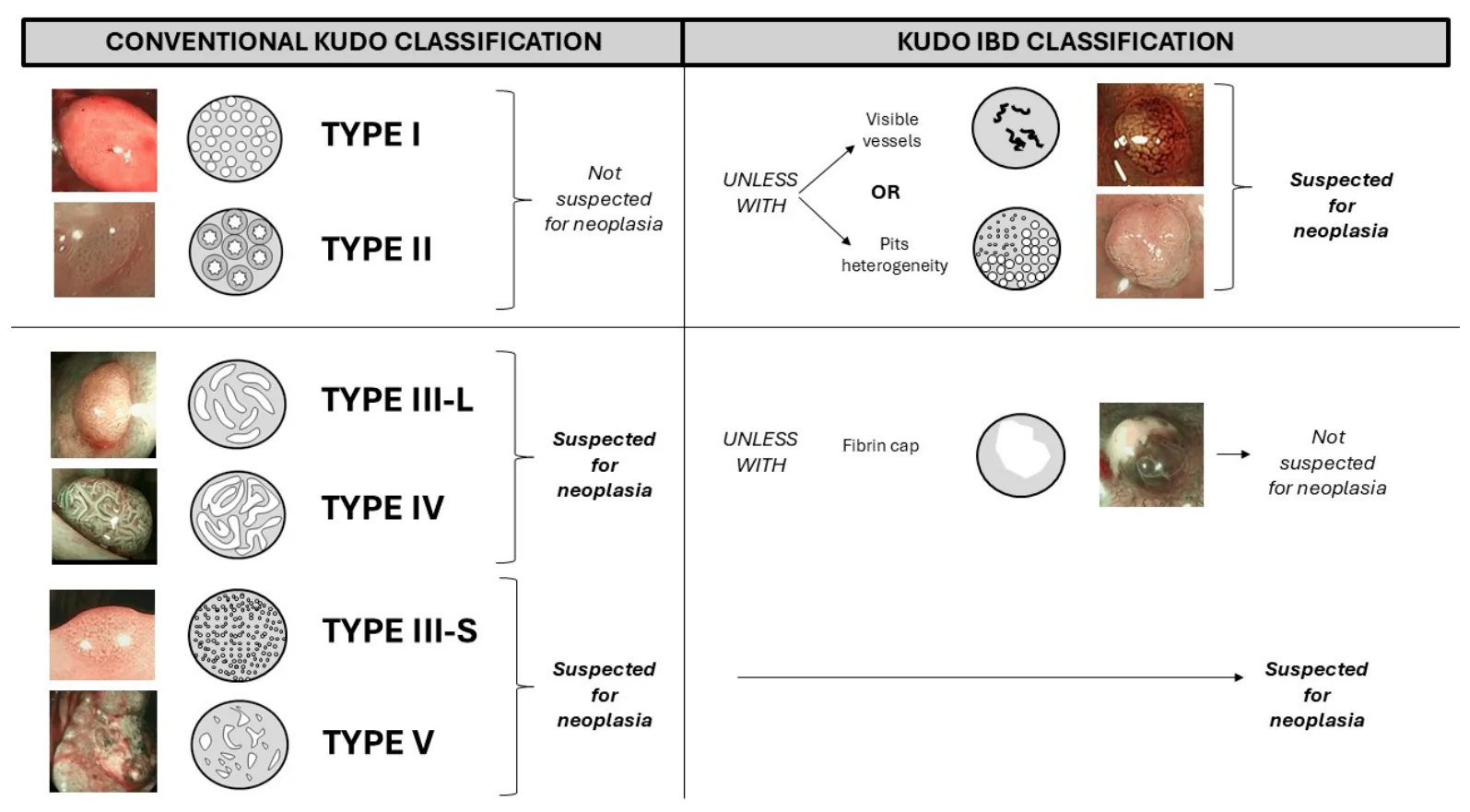
Kudo and Kudo-IBD classifications for lesion characterization.

According to the original Kudo classification, patterns I-II indicate non-neoplastic lesions, whereas patterns III-L, IV, III-S, and V indicate neoplastic lesions [5]. According to the Kudo-IBD classification (Figure 2), Kudo I-II patterns indicate neoplastic lesions if they have at least one of two additional endoscopic factors: (1) visible vessels and (2) pits heterogeneity; in contrast, Kudo III-IV or unclassified “0” patterns are suspected non-neoplastic if there is associated fibrin cap. Finally, Kudo V and III-S patterns always indicate neoplastic lesions [4].

According to the NICE classification, *type-1* lesions indicate non-neoplastic lesions, while *type-2* and *type-3,* which are associated with adenoma and invasive cancer respectively, indicate neoplastic lesions [6].

### 2.5. Artificial Intelligence

CAD-EYE was used according to the manufacturer’s recommendations in both computer-aided detection (CADe) and computer-aided diagnosis (CADx) modes.

CAD-EYE is activated by pressing a button on the endoscope’s handle and provides both a sound signal and a visual assist square when a suspected lesion is detected. Importantly, because CAD-EYE was activated only during the final withdrawal after completion of WLI and LCI examinations, lesion detection by the AI system represented the autonomous output of the detection algorithm rather than an additional subjective evaluation by the operator. Consequently, although operators were aware of previously detected lesions, CAD-EYE alerts were generated automatically by the software based on real-time image analysis rather than on the operator’s subjective decision to activate or interpret a previously identified lesion.

After lesion detection, BLI was activated to support CAD-EYE characterization as suspected neoplastic or non-neoplastic. Notably, although CAD-EYE has demonstrated excellent performance for colorectal lesion detection and characterization in average-risk populations, the system was not specifically trained using UC-associated neoplasia. Consequently, the present study prospectively evaluated its performance in the setting of chronic inflammatory bowel disease.

### 2.6. Histopathological Assessment

All lesions underwent biopsy or endoscopic resection after completion of the optical evaluation according to routine clinical practice. Histological sampling or endoscopic resection was intentionally postponed until completion of the entire optical assessment to avoid bleeding or mucosal alterations potentially affecting subsequent evaluation.

Histopathological examination represented the reference standard for all analyses. Specimens were independently evaluated by three experienced gastrointestinal pathologists, who were blinded to the optical diagnosis and CAD-EYE predictions. Specimens were fixed in formalin and analyzed histologically according to guidelines [18].

### 2.7. Endpoints

For each withdrawal (WLI, LCI, CAD-EYE), detection outcomes included the following:

- The number of lesions found (*lesion detection rate*);
- The number of neoplastic lesions found (*neoplasia detection rate*);
- The number of lesions “missed”, i.e., not identified by each technology when compared with the other technologies (*lesion miss rate*);
- The number of neoplastic lesions “missed” according to the same criteria (*neoplasia miss rate*);
- The visibility score of each lesion, considered good when at least 3 points were assigned.

The outcomes for optical characterization included the sensitivity, specificity, positive predictive value (PPV), negative predictive value (NPV) and overall diagnostic accuracy of CAD-EYE; the Kudo pit pattern classification; the NICE classification; and the Kudo-IBD classification for the prediction of colorectal neoplasia.

Exploratory analyses evaluated the influence of local inflammatory activity according to the Mayo Endoscopic Subscore (MES), lesion size, anatomical location, Paris morphology, and other recorded lesion characteristics on diagnostic performance.

### 2.8. Sample Size and Statistical Analyses

At the time of study conception, no previous studies had reported the use of ELUXEO chromoendoscopy or CAD-EYE AI for the detection and characterization of lesions in patients with UC. Therefore, the present study was designed as a pilot study.

At least 100 lesions were required for lesion characterization. Based on previous studies reporting 3–5 lesions per patient [7,8], at least 40 patients were required.

For lesion detection, a previous study in the general population estimated that 278 polyps were required to detect a 15% difference between LCI and WLI with 90% power and α = 0.05 [19]. Assuming four lesions per patient, at least 70 patients were therefore required.

Continuous variables were reported as mean ± standard deviation or median (interquartile range), according to data distribution. Categorical variables were expressed as frequencies and percentages. Exact 95% confidence intervals (C.I.) were calculated using the Clopper–Pearson method. Paired detection modalities were compared using exact McNemar’s tests. Sensitivity, specificity, and overall diagnostic accuracy were compared between paired characterization methods using exact McNemar’s tests. PPV and NPV were reported descriptively and were not formally compared between methods.

For prespecified exploratory subgroup analyses, global comparisons of paired detection modalities and characterization accuracy were performed using Cochran’s Q test. These analyses were considered exploratory and were interpreted descriptively.

Prespecified exploratory analyses also evaluated whether lesion characteristics were associated with diagnostic performance. The characteristics examined included local MES, anatomical location, Paris morphology, lesion colour, pit-pattern heterogeneity, presence of defined vessels, fibrin, surface inflammation, and lesion size. For each characterization method, sensitivity, specificity, PPV, NPV, and overall accuracy were evaluated separately according to the categories of each lesion characteristic. Associations involving categorical characteristics were assessed using Fisher’s exact test or the chi-square test, as appropriate, whereas lesion size was analyzed using the Mann–Whitney U test. To account for multiple testing, *p* values from the lesion-characteristic analyses were adjusted using the Benjamini–Hochberg false discovery rate procedure. Lesion size was considered exploratory because of the limited number of lesions in some size strata.

All primary diagnostic performance analyses were performed at the lesion level. Because multiple lesions could be identified in individual patients, observations were not necessarily independent. No specific patient-level clustering adjustment was applied in the present exploratory analysis; this limitation was taken into account when interpreting statistical precision and generalizability.

All statistical tests were two-sided. For the primary analyses, a *p* value < 0.05 was considered statistically significant, whereas statistical significance for the exploratory lesion-characteristic analyses was defined by an adjusted *p* value (*q* value) < 0.05. Diagnostic accuracy analyses were based on the prospectively recorded final classification/suspicion variables. Statistical analyses were performed using IBM SPSS Statistics version 24 (IBM Corp., Armonk, NY, USA) and Microsoft Excel 2016 (Microsoft Corporation, Redmond, WA, USA). All authors had access to the study data and reviewed and approved the final manuscript.

### 2.9. Measures to Minimize Bias

Several methodological measures were adopted to reduce potential sources of bias. Consecutive patients were prospectively enrolled according to predefined eligibility criteria, and all examinations followed a standardized sequential protocol. Endoscopists underwent protocol calibration before study initiation. Histopathology served as the reference standard, and gastrointestinal pathologists remained blinded to all endoscopic optical diagnoses and AI predictions.

The tandem colonoscopy design inevitably precluded complete blinding between sequential withdrawal phases, and some degree of recognition bias cannot therefore be completely excluded. However, this limitation mainly affects comparisons between operator-dependent imaging modalities. Importantly, CAD-EYE remained deactivated during the WLI and LCI examinations and was activated only during the final withdrawal. Therefore, lesion detection during the AI-assisted phase represented the autonomous output of the artificial intelligence algorithm, which generated real-time alerts independently of the endoscopist’s subjective decision to activate or interpret a previously identified lesion. Nevertheless, the fixed examination order and the absence of prospectively recorded withdrawal times precluded complete separation of the effect of CAD-EYE from potential sequence- and time-related effects.

Withdrawal times were not prospectively recorded because the study was conceived as a pragmatic clinical investigation. The sequential examination protocol included image acquisition and lesion documentation during the procedure, whereas optical characterization and tissue sampling or endoscopic resection were performed after completion of the three withdrawal phases. Consequently, reliable attribution of examination time to individual imaging modalities was not possible.

## 3. Results

### 3.1. Patients

From February 2022 to February 2024, 84 patients were enrolled, two of whom were excluded due to concurrent primary sclerosing cholangitis. Therefore, 82 patients were analyzed (Table 1). No missing data were observed for the primary study outcomes. Therefore, no imputation procedures were required and all analyses were performed on complete cases. Males and females were equally distributed, with mean age at examination of 56 years and a mean disease duration of 18 years. Most patients were in clinical remission; however, endoscopic disease activity was detected in almost half of colonoscopies.

### 3.2. Lesions

In 54 patients (66%), 281 lesions were identified (mean 5/patient) (Table 2). Most lesions were small (mean 6 mm, range 2–15 mm) and sessile (I-s: 79%) lesions, with only six (2%) laterally spreading lesions (LSLs).

The majority of lesions were detected in the rectum and sigmoid, while around one third were found in the right colon.

Histologically, most lesions were inflammatory (79%), while 13% were hyperplastic lesions; no sessile serrated lesions (SSLs) without dysplasia or traditional serrated adenomas were found. In 19 patients (23% of total patients, 35% of those with at least one visible lesion), 22 neoplastic lesions (8% of total lesions) were found, of which almost all were lesions with low-grade dysplasia, including one dysplastic SSL with low-grade dysplasia (LGD). Only one lesion with high-grade dysplasia was found, while no adenocarcinoma was found.

Morphologically, neoplastic lesions were typically small (mean 6 mm, range 2–15 mm) poorly elevated lesions (II-a: 64%), located in the right colon (73%).

### 3.3. Lesion Detection

Of the 281 lesions found, 16 lesions (5.7%) were missed by at least one method. In general, missed lesions were small (mean 5 mm, range 2–8 mm), poorly elevated (II-a = 8), sessile (Is = 6) or flat (II-b = 2), and located mainly in the right colon (cecum = 2, ascending = 8, transverse = 3, descending = 1, sigmoid = 2, rectum = 1). Four missed lesions were neoplastic (including a 7 mm dysplastic SSL in the ascending colon, type IIa), four were hyperplastic, and eight were inflammatory.

**Figure 3:**
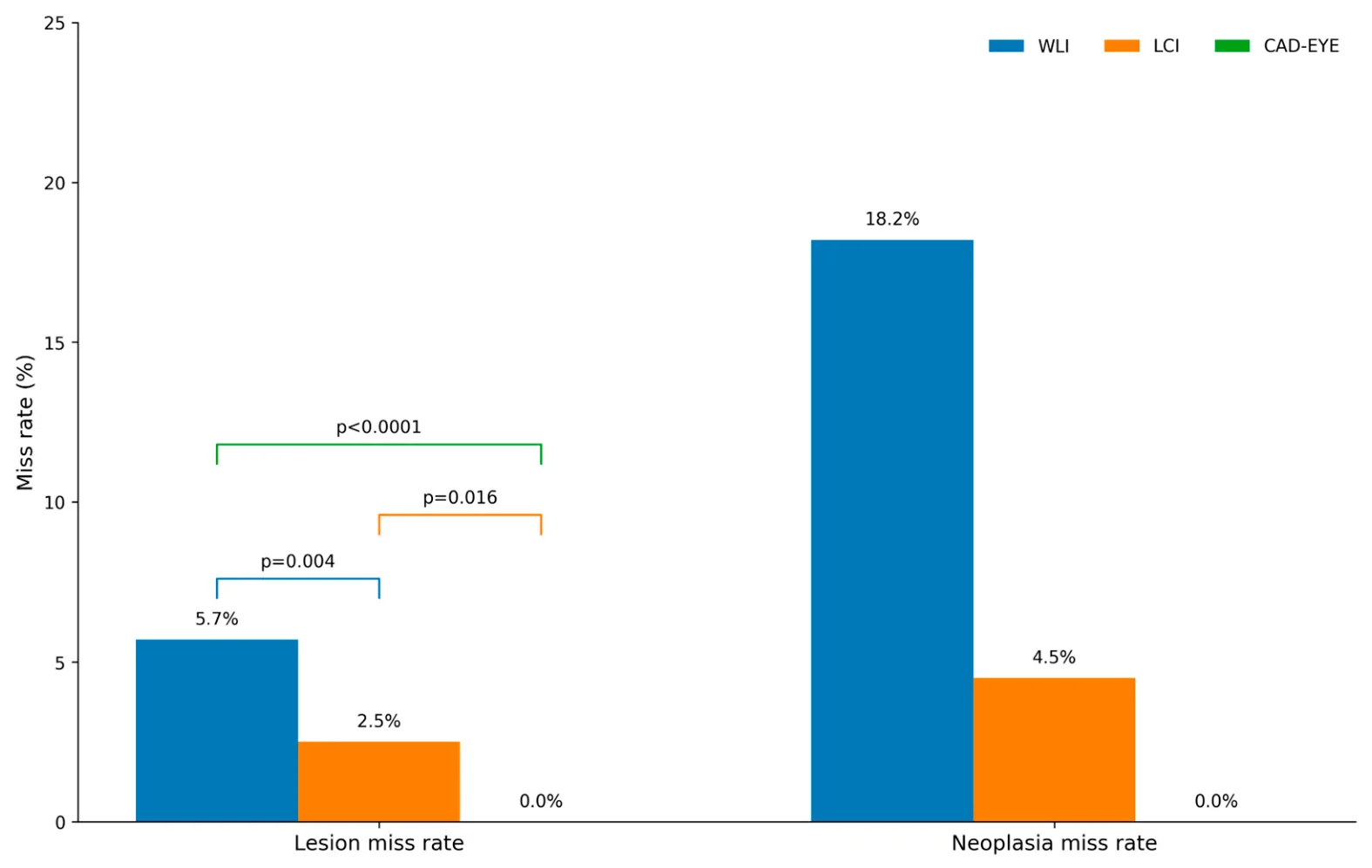
Lesion and neoplasia miss rates during the detection phase. WLI = white-light imaging; LCI = linked-colour imaging.

The lesion miss rate was significantly higher with WLI (n = 16/281 [5.7%; 95% C.I. 3.5–9.1]) than LCI (n = 7 [2.5%; 95% C.I. 1.2–5.0]; exact McNemar *p* = 0.004). No lesions were missed during the CAD-EYE-assisted withdrawal, resulting in a miss rate of 0% (95% C.I. 0.0–1.35) and a detection rate of 100% (95% C.I. 98.7–100). Compared with WLI, CAD-EYE significantly reduced lesion miss rate (*p* < 0.0001) and also outperformed LCI (*p* = 0.016) (Figure 3).

The neoplasia miss rate was highest with WLI (n = 4/22; 18.2%), whereas one adenoma was missed with LCI (4.5%) and none with CAD-EYE. However, pairwise comparisons did not reach statistical significance (WLI vs. LCI *p* = 0.250; WLI vs. CAD-EYE *p* = 0.125; LCI vs. CAD-EYE *p* = 1.000) (Figure 3).

Mean visibility scores were 3.68 with WLI, 3.81 with LCI, and 3.99 with CAD-EYE. Poor visibility (visibility score < 3) was observed in 20 lesions with WLI (7.1%), 10 with LCI (3.6%), and one with CAD-EYE (0.4%). Compared with CAD-EYE, poor visibility was significantly more frequent with WLI (*p* < 0.001) and LCI (*p* = 0.004).

### 3.4. Lesion Characterization

Detailed diagnostic performance data are summarized in Table 3. Kudo-IBD showed the highest diagnostic performance across the evaluated metrics, with a sensitivity of 90.9%, specificity of 85.3%, PPV of 34.5%, NPV of 99.1%, and overall accuracy of 85.8%. CAD-EYE showed a sensitivity of 77.3%, specificity of 73.7%, PPV of 20.0%, NPV of 97.4%, and accuracy of 74.0%. The corresponding estimates for KUDO were 63.6%, 74.9%, 17.7%, 96.0%, and 74.0%, respectively, whereas NICE showed a sensitivity of 68.2%, specificity of 64.1%, PPV of 13.9%, NPV of 96.0%, and accuracy of 64.4%.

**Figure 4:**
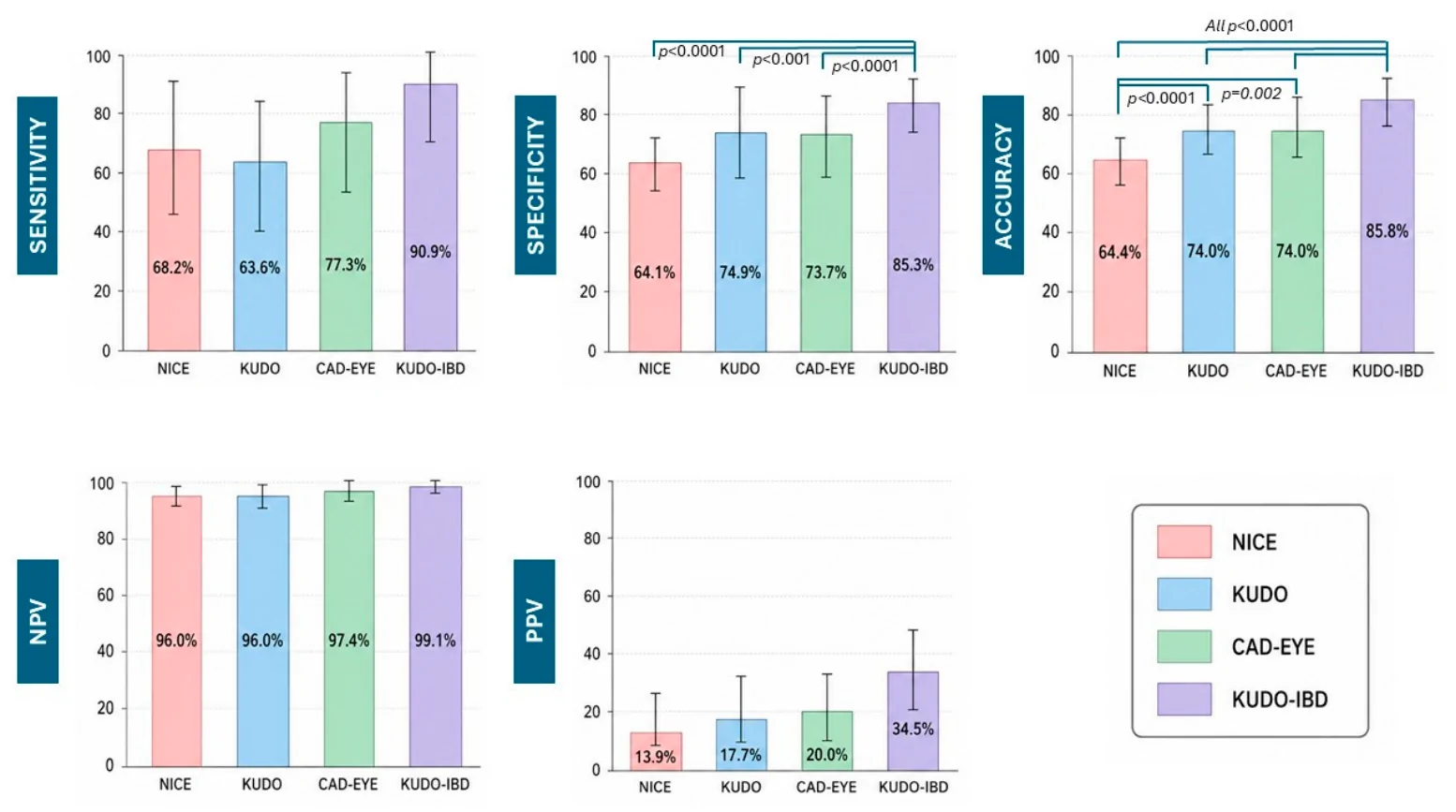
Diagnostic accuracy of endoscopic classifications and artificial intelligence in lesion characterization. PPV = positive predictive value. NPV = negative predictive value.

As shown in Figure 4, Kudo-IBD showed the highest sensitivity, although pairwise differences in sensitivity were not statistically significant. In contrast, Kudo-IBD demonstrated significantly higher specificity than CAD-EYE, Kudo, and NICE (all *p* < 0.001). Kudo-IBD also showed significantly higher overall accuracy than CAD-EYE, Kudo, and NICE (all *p* < 0.001). NICE showed significantly lower accuracy than CAD-EYE, Kudo, and Kudo-IBD (*p* < 0.01 for all comparisons), whereas no significant difference was observed between CAD-EYE and Kudo.

Kudo-IBD also showed the highest PPV (34.5%) compared with CAD-EYE (20.0%), Kudo (17.7%), and NICE (13.9%); however, PPV was reported descriptively and was not formally compared between methods. By contrast, NPV was consistently high across all four approaches, ranging from 96.0% for Kudo and NICE to 99.1% for Kudo-IBD.

### 3.5. False-Positive and False-Negative Diagnoses

False-positive diagnoses represented the main source of diagnostic error across all evaluated methods (Table 3). NICE generated the highest number of false-positive classifications (93/259 non-neoplastic lesions), followed by CAD-EYE (68/259), Kudo (65/259), and Kudo-IBD (38/259). False-negative diagnoses were less frequent overall. Kudo-IBD yielded the lowest number of false-negative classifications (2/22 neoplastic lesions), compared with CAD-EYE (5/22), NICE (7/22), and Kudo (8/22).

### 3.6. Influence of Lesion Characteristics on Detection and Characterization

Exploratory analyses evaluated the influence of lesion characteristics on detection and optical characterization.

CAD-EYE detected all lesions across all evaluated subgroups. Detection performance nevertheless differed between WLI, LCI, and CAD-EYE according to local inflammatory activity, lesion size, anatomical location, and Paris morphology. Detailed subgroup comparisons are reported in Supplementary Tables S1–S4.

For optical characterization, several lesion characteristics were significantly associated with diagnostic performance after Benjamini–Hochberg correction for multiple testing (Table 4). These associations were observed predominantly for specificity and overall accuracy, whereas associations with sensitivity were less frequent. Across methods, local inflammatory activity, anatomical site, lesion colour, pit-pattern heterogeneity, fibrin, surface inflammation, and the presence of visible vessels were among the characteristics most frequently associated with diagnostic performance.

The pattern of associations differed between characterization methods (Table 4). CAD-EYE showed significant associations between lesion characteristics and specificity, PPV, NPV, and accuracy, but no significant association with sensitivity. Kudo-IBD showed significant associations with specificity, PPV, and accuracy, whereas sensitivity and NPV were not significantly associated with the evaluated characteristics. Kudo and NICE showed broader patterns of association across several diagnostic parameters, particularly specificity and accuracy.

Kudo-IBD maintained the highest overall characterization accuracy across the prespecified subgroup analyses. Accuracy was 85.8% for lesions <10 mm and 85.0% for lesions ≥10 mm, 83.6% for right-sided and 89.1% for left-sided lesions, and 85.8% for polypoid and 83.6% for non-polypoid lesions. Global differences in characterization accuracy were statistically significant for MES 0, MES 1, and MES 3. Similarly, significant global differences were observed according to lesion size, right-sided location, and both polypoid and non-polypoid morphology, whereas the comparison for left-sided lesions was not statistically significant.

Given the exploratory nature of these analyses, the limited number of neoplastic lesions, and the multiple comparisons performed, these findings should be interpreted as descriptive and hypothesis-generating rather than as evidence of independent determinants of diagnostic performance.

## 4. Discussion

In this prospective tandem colonoscopy study of 281 lesions detected during surveillance of 82 patients with UC, the combination of CAD-EYE artificial intelligence with LCI/BLI virtual chromoendoscopy was superior for lesion detection, whereas disease-specific optical diagnosis using Kudo-IBD provided the best characterization performance. Three main clinical messages can therefore be drawn.

First, AI-assisted colonoscopy using CAD-EYE can improve lesion detection. This finding is consistent with previous tandem colonoscopy studies in the general population reporting reduced adenoma miss rates with AI compared with HD-WLE [20,21,22]. On the other hand, evidence regarding AI-assisted detection in IBD remains limited. Most commercially available systems have been developed using sporadic colorectal lesions in non-inflamed mucosa, whereas UC-associated dysplasia develops in a substantially different inflammatory and endoscopic environment. Nonetheless, our study shows that CAD-EYE maintained complete lesion detection across all evaluated subgroups, including different degrees of local inflammation, lesion sizes, anatomical locations, and Paris morphologies.

The clinical significance of this finding should nevertheless be interpreted cautiously. Because CAD-EYE was always used after WLI and LCI, and withdrawal times were not prospectively recorded, these findings do not establish that the observed improvement was attributable independently to CAD-EYE. Moreover, the absolute number of neoplastic lesions was limited, and the difference in neoplasia miss rate did not reach statistical significance. Finally, most lesions missed by WLI and LCI were small and non-neoplastic. Thus, our findings support CAD-EYE as a highly sensitive lesion-finding adjunct, but do not by themselves demonstrate a reduction in dysplasia-related risk or interval colorectal cancer.

Second, lesion characterization remained substantially more dependent on disease-specific optical assessment. Kudo-IBD achieved the highest overall diagnostic performance, with a sensitivity of 90.9%, specificity of 85.3%, and overall accuracy of 85.8%, and its accuracy was significantly higher than that of CAD-EYE, Kudo, and NICE. These findings support the concept that classifications specifically developed for IBD may better account for the architectural and vascular alterations associated with chronic inflammation than classifications originally designed for sporadic colorectal neoplasia.

An additional clinically relevant observation was the high frequency of false-positive diagnoses. False-positive classifications represented the main source of diagnostic error across all four methods. The exploratory lesion-characteristic analysis identified local inflammatory activity and surface inflammation among the features significantly associated with diagnostic performance, particularly specificity, PPV, and overall accuracy, suggesting that inflammatory changes may contribute to diagnostic uncertainty during UC surveillance. NICE generated the highest number of false-positive classifications, whereas Kudo-IBD substantially reduced false-positive diagnoses while maintaining the highest sensitivity. Conversely, CAD-EYE, despite its excellent performance for lesion detection, showed lower specificity when used for optical characterization. These findings reinforce the distinction between lesion detection and lesion characterization as two different diagnostic tasks.

The exploratory analysis of lesion characteristics provides an additional perspective on optical characterization. Diagnostic performance varied according to several lesion characteristics, although the pattern differed among methods and diagnostic parameters (Table 4). After correction for multiple testing, associations were identified for local inflammatory activity, anatomical site, Paris morphology, lesion colour, pit-pattern heterogeneity, visible vessels, fibrin cap, surface inflammation, and LSL morphology. These associations were driven by specificity, PPV, NPV, and overall accuracy considerably more frequently than sensitivity. The findings therefore suggest that the ability of optical characterization systems to correctly classify lesions is influenced by the morphological and inflammatory context in which they are applied.

This observation is clinically plausible in UC surveillance. Chronic inflammation, regenerative change, altered vascularity, and architectural distortion may reproduce optical features traditionally associated with neoplasia, increasing the overlap between neoplastic and non-neoplastic mucosa. The frequent associations between inflammatory or morphological features and specificity, PPV, and accuracy may therefore help explain why false-positive characterization remains a major source of diagnostic error in this setting. Importantly, however, these associations should not be interpreted as independent causal effects of individual lesion characteristics, because the evaluated features are biologically and morphologically interrelated.

The pattern of associations also differed among the four characterization methods. NICE and Kudo showed associations with a relatively broad range of lesion characteristics, particularly involving specificity, PPV, NPV, and accuracy. CAD-EYE similarly showed associations with several lesion characteristics, including local inflammatory activity, anatomical site, lesion colour, pit-pattern heterogeneity, fibrin cap, and surface inflammation. Kudo-IBD showed a narrower pattern, with significant associations mainly involving specificity, PPV, and accuracy, whereas sensitivity was not significantly associated with the evaluated characteristics after correction for multiple testing. This may suggest greater robustness of Kudo-IBD for identifying neoplastic lesions across different endoscopic phenotypes, although this interpretation remains preliminary because only 22 neoplastic lesions were available for analysis.

However, these subgroup analyses and the lesion-characteristic associations should be considered exploratory. Several strata contained few or no neoplastic lesions, and the number of statistical comparisons was substantial. Although the Benjamini–Hochberg procedure was applied to control the false discovery rate, the analyses were not designed to establish independent predictors of diagnostic performance. The findings should therefore be regarded as hypothesis-generating and require validation in larger cohorts.

The relatively low PPV observed with all four approaches should be interpreted in the context of the low prevalence of neoplasia in the study population (7.8%). PPV is strongly dependent on disease prevalence and therefore cannot be interpreted independently of the underlying case mix. Conversely, NPV was high across all four methods, ranging from 96.0% for Kudo and NICE to 99.1% for Kudo-IBD. In this cohort, a negative optical diagnosis was therefore rarely associated with underlying neoplasia.

Kudo-IBD showed the highest PPV (34.5%), compared with 20.0% for CAD-EYE, 17.7% for Kudo, and 13.9% for NICE. However, PPV was reported descriptively and was not formally compared between methods. Consequently, these differences should be considered descriptive rather than evidence of statistically demonstrated superiority. The same caution applies to PPV findings in the exploratory lesion-characteristic analyses.

These findings may nevertheless have potential implications for optical management strategies. In lesions with optical features strongly suggestive of neoplasia, Kudo-IBD may provide useful real-time characterization to support selection of the most appropriate endoscopic treatment. Conversely, lesions showing a Kudo-I pattern without additional suspicious features may potentially be considered for a diagnose-and-leave approach, whereas uncertain lesions should remain candidates for targeted biopsy [23]. These implications, however, require prospective validation specifically in IBD populations before changes in clinical management can be recommended.

Our findings should also be considered in the context of the limited available evidence on AI-assisted colonoscopy in IBD. Only a limited number of studies have evaluated AI systems in IBD surveillance [24,25,26,27,28,29], and the available evidence remains heterogeneous. Lopez-Serrano et al. reported similar dysplasia detection rates between i-SCAN VCE and the Discovery AI system in 48 lesions, although specificity and PPV were low [24]. Abdelrahim et al. described a novel AI model validated in 25 lesions, with sensitivity of 87.5% and specificity of 80.6% for lesion characterization [25]. Picardo et al. evaluated CAD-EYE in 97 lesions from patients with IBD, reporting a sensitivity of 80% and specificity of 97.6% [29]. Although sensitivity and NPV appear broadly comparable to those reported in non-IBD series, PPV appears substantially lower in IBD [10,11,12,13]. Differences among studies may reflect differences in lesion prevalence, histological composition, and, particularly, the criteria used to classify inflammatory pseudopolyps. Our study adds a direct comparison of AI-assisted detection and several optical characterization systems within the same lesion set and suggests that, in UC surveillance, the principal value of CAD-EYE lies in lesion detection, whereas disease-specific optical assessment remains more appropriate for characterization.

The main conceptual implication of our study is therefore that artificial intelligence and disease-specific optical diagnosis should be considered complementary rather than competing technologies. CAD-EYE performed optimally during lesion detection, functioning as a computer-assisted observer that continuously analyses the endoscopic image and generates automated alerts. This may be particularly useful during surveillance examinations, in which numerous small lesions and subtle mucosal abnormalities must be identified and operator fatigue may contribute to missed lesions. Once a lesion has been identified, however, Kudo-IBD provides a more accurate disease-specific framework for real-time characterization and therapeutic decision-making. A multimodal surveillance strategy could therefore combine AI-assisted detection with expert disease-specific optical characterization.

The tandem colonoscopy design deserves specific consideration. Although not randomized by definition, tandem colonoscopy is widely used to evaluate endoscopic technologies because each patient serves as their own control [15,16,30,31]. Sequential withdrawal nevertheless introduces the possibility of recognition bias because lesions identified during earlier examination phases may be more easily recognized during subsequent examinations. Several methodological measures were implemented to minimize this limitation. Tissue sampling was systematically postponed until completion of optical assessment, thereby preserving mucosal integrity throughout the examination. Furthermore, CAD-EYE remained deactivated during the WLI and LCI withdrawals and was activated exclusively during the final withdrawal. Consequently, lesion recognition during the AI-assisted phase represented the autonomous output of the detection algorithm rather than an additional subjective evaluation by the endoscopist.

Our study has several limitations. First, it was conducted at a single tertiary referral centre by experienced IBD endoscopists, which may limit generalisability to less specialized settings. Second, although the prospective design strengthens internal validity, only 22 neoplastic lesions were identified, limiting statistical precision and preventing robust subgroup analyses. Adenocarcinoma and SSL without dysplasia were also not observed, precluding assessment of diagnostic performance in these lesions. Third, withdrawal times were not prospectively recorded, preventing reliable assessment of whether differences in examination duration contributed to the observed detection rates. Fourth, the fixed examination sequence, with WLI always performed before LCI and CAD-EYE-assisted imaging, may have introduced sequence-related effects, including increased operator familiarity with lesion locations and recognition bias. Although CAD-EYE generated automated alerts based on real-time image analysis, the operators were aware of lesions identified during previous phases; therefore, the independent incremental contribution of CAD-EYE cannot be determined. Fifth, analyses were performed at the lesion level, although multiple lesions could occur within the same patient. No specific adjustment for intra-patient clustering was applied; consequently, estimates of statistical precision and associated significance tests should be interpreted cautiously. Finally, CAD-EYE was not specifically trained on UC-associated neoplasia, and its characterization performance may differ in populations with different inflammatory and neoplastic profiles.

The exploratory analysis of lesion characteristics represents an additional limitation. Although multiple testing was addressed using the Benjamini–Hochberg false discovery rate procedure, the relatively small number of neoplastic lesions and the interdependence among lesion characteristics limit the stability and generalizability of individual associations. In particular, estimates of sensitivity and PPV within specific phenotypic strata are inherently imprecise. These analyses should therefore be considered hypothesis-generating and require external validation before being used to define phenotype-specific characterization algorithms or management pathways.

Despite these limitations, the study has several important strengths. To our knowledge, this is one of the first prospective studies to evaluate lesion detection and optical characterization simultaneously within the same structured surveillance protocol in UC. All lesions underwent histopathological confirmation, examinations followed a standardized imaging sequence, and histopathological assessment was performed by blinded gastrointestinal pathologists. Importantly, rather than evaluating isolated technologies, the study investigated how advanced imaging, artificial intelligence, and disease-specific optical diagnosis can be integrated into a clinically applicable surveillance pathway, thereby reflecting real-world endoscopic practice.

Future multicentre studies including larger cohorts and a greater number of dysplastic lesions are warranted to validate these findings. In particular, development of artificial intelligence systems specifically trained on UC-associated dysplasia may improve optical characterization and further optimize computer-assisted surveillance in IBD. Future studies should also determine whether integration of AI-assisted detection with disease-specific optical classification translates into improved clinical outcomes, including reduced interval colorectal cancer and fewer unnecessary biopsies.

## 5. Conclusions

In conclusion, our findings suggest that AI-assisted detection and disease-specific optical characterization provide complementary benefits at different stages of surveillance colonoscopy. CAD-EYE may improve lesion detection during surveillance colonoscopy in patients with UC, whereas the disease-specific KUDO-IBD classification provided the highest diagnostic accuracy for optical characterization. Moreover, exploratory analyses indicate that lesion characteristics influence optical characterization performance, particularly specificity, PPV, NPV, and overall accuracy, while CAD-EYE maintained complete lesion detection across the evaluated phenotypic subgroups. Rather than representing competing approaches, these technologies may therefore be integrated sequentially, with AI supporting lesion detection and disease-specific optical assessment guiding subsequent characterization. These findings support a multimodal surveillance strategy in which AI enhances lesion detection while expert disease-specific optical assessment guides real-time characterization and therapeutic decision-making. Larger studies using independent or counterbalanced examination sequences and prospectively recorded withdrawal times are needed to clarify the incremental contribution of CAD-EYE. Further external validation of the Kudo-IBD system is also warranted.

## Figures and Tables

**Table 1:** Characteristics of patients at enrolment.

Variables	Data
Sex	Male = 46 (56%)
	Female = 36 (44%)
Age—mean	56 years (range 18–80 years)
Disease duration—mean	18 years (range 8–39 years)
Disease extension—n (%)	Proctitis: 5 (6%)
	Left colitis: 28 (34%)
	Extensive colitis: 49 (60%)
Previous dysplasia—n (%)	13 (16%)
Previous pseudopolyps—n (%)	14 (17%)
Family history of CRC—n (%)	7 (9%)
Active smokers—n (%)	8 (10%)
Clinical activity—n (%)	15 (18%)
Endoscopic activity—n (%)	34 (41%)
	Mayo 1: 12 (15%)
	Mayo 2: 9 (11%)
	Mayo 3: 13 (16%)

**Table 2:** Characteristics of lesions detected during the study.

Variables	All Lesions (n = 281)	Neoplastic Lesions (n = 22)
Site—n (%)	Rectum: 50 (18%) Sigmoid: 89 (32%) Descending: 50 (18%) Transverse: 53 (19%) Ascending: 35 (12%) Cecum: 4 (1%)	Rectum: 1 (4%) Sigmoid: 4 (18%) Descending: 1 (4%) Transverse: 6 (27%) Ascending: 8 (36%) Cecum: 2 (9%)
Size—mean	6 mm (range 2–15 mm)	6 mm (range 2–15 mm)
Morphology (Paris classification)—n (%)	Is: 223 (79%) Isp: 1 (0.4%) Ip: 3 (1%) IIa: 47 (17%) IIb: 7 (2.5%) IIc: 0 (0%) 0-III: 0 (0%)	Is: 6 (27%) Isp: 0 (0%) Ip: 1 (4%) IIa: 14 (64%) IIb: 1 (4%) IIc: 0 (0%) 0-III: 0 (0%)
Histology	Neoplastic: 22 (8%) Hyperplasic: 36 (13%) Inflammatory: 222 (79%) Lipoma: 1 (0.3%)	Low-grade dysplasia: 20 (37%) High-grade dysplasia: 1 (2%) Sessile serrated lesion with dysplasia: 1 (2%) Adenocarcinoma: 0 (0%)

**Table 3:** Diagnostic performance of the four optical characterization methods. TP = true positives. FP = false positives. FN = false negatives. TN = true negatives. PPV = positive predictive values. NPV = negative predictive values. Values of each metrics are reported as % (95% C.I.).

Method	TP	FP	FN	TN	Sensitivity	Specificity	PPV	NPV	Accuracy
**CAD-EYE**	17	68	5	191	77.3 (54.6–92.2)	73.7 (67.9–79.0)	20.0 (12.1–30.1)	97.4 (94.1–99.2)	74.0 (68.5–79.0)
**Kudo-IBD**	20	38	2	221	90.9 (70.8–98.9)	85.3 (80.4–89.4)	34.5 (22.5–48.1)	99.1 (96.8–99.9)	85.8 (81.1–89.6)
**Kudo**	14	65	8	194	63.6 (40.7–82.8)	74.9 (69.2–80.1)	17.7 (10.0–27.9)	96.0 (92.3–98.3)	74.0 (68.5–79.0)
**NICE**	15	93	7	166	68.2 (45.1–86.1)	64.1 (57.9–69.9)	13.9 (8.0–21.9)	96.0 (91.8–98.4)	64.4 (58.5–70.0)

**Table 4:** Lesion characteristics associated with diagnostic performance. Sens = sensitivity. Sp = specificity. PPV = positive predictive value. NPV = negative predictive value. Acc = overall accuracy. MES = Mayo Endoscopic Subscore. LSL = laterally spreading lesion. Associations were evaluated separately for each diagnostic parameter and characterization method. Categorical characteristics were assessed using Fisher’s exact test or the chi-square test, as appropriate, whereas lesion size was analyzed using the Mann–Whitney U test. *p* values were adjusted using the Benjamini–Hochberg false discovery rate procedure. Only associations with adjusted *q* < 0.05 are shown. Lesion size was considered exploratory and is reported separately in the Supplementary Materials.

Lesion Characteristic	NICE	KUDO	CAD-EYE	KUDO-IBD
**Local MES**	Sp, PPV, Acc	Sp, PPV, Acc	Sp, PPV, Acc	Sp, Acc
**Anatomical site**	Sp, PPV, NPV, Acc	Sp, PPV, NPV, Acc	Sp, PPV, Acc	Sp, PPV, Acc
**Paris morphology**	PPV, NPV	PPV, NPV	Sp, PPV, NPV, Acc	PPV
**LSL**	Sp, Acc	Sp, Acc	—	—
**Dark colour**	Sp, PPV, Acc	Sp, PPV, Acc	Sp, Acc	Sp, Acc
**Pit-pattern heterogeneity**	Sens, Sp, PPV, NPV, Acc	Sp, NPV, Acc	Sp, Acc	Sp, Acc
**Visible vessels**	PPV, NPV	PPV, NPV	PPV	Sp, PPV
**Fibrin cap**	Sp, PPV, Acc	Sp, PPV, Acc	Sp, PPV, Acc	—
**Surface inflammation**	Sp, PPV, Acc	Sp, PPV, Acc	Sp, PPV, Acc	Sp, PPV, Acc

## Data Availability

The data that support the findings of this study are available from the corresponding author upon reasonable request.

## Supplementary Materials

The following supporting information can be downloaded at https://www.mdpi.com/article/10.3390/jcm15187290/s1: Table S1: Lesion detection and characterization performance according to local inflammatory activity (MES). Table S2: Lesion detection and characterization performance according to lesion size. Table S3: Lesion detection and characterization performance according to anatomical location. Table S4: Lesion detection and characterization performance according to Paris morphology.
