# Supplementary material for: Complementary Roles of Artificial Intelligence and Disease-Specific Optical Diagnosis During Ulcerative Colitis Surveillance: A Prospective Tandem Colonoscopy Study
Source: J Clin Med. 2026 Sep 19;15(18):7290. doi: 10.3390/jcm15187290 (PMC13607791; doi:10.3390/jcm15187290)
Supplement: Supplementary file 1 [file jcm-15-07290-s001.zip › jcm-4325861-supplementary.pdf]

Supplementary Table S1. Lesion detection and characterization performance according to local inflammatory activity (MES).

Subgroup	n	Neoplastic	WLI miss (95% CI)	LCI miss (95% CI)	CAD-EYE miss (95% CI)	*P* Detection	CAD-EYE Accuracy	KUDO-IBD Accuracy	KUDO Accuracy	NICE Accuracy	*P* Characterization
MES 0	188	20	7.4% (4.1–12.2)	3.7% (1.5–7.5)	0.0% (0.0–1.9)	**<0.001**	78.2% (71.6–83.9)	86.7% (81.0–91.2)	86.2% (80.4–90.8)	74.5% (67.6–80.5)	**<0.001**
MES 1	25	0	4.0% (0.1–20.4)	0.0% (0.0–13.7)	0.0% (0.0–13.7)	**0.368**	84.0% (63.9–95.5)	100.0% (86.3–100.0)	80.0% (59.3–93.2)	60.0% (38.7–78.9)	**<0.001**
MES 2	14	2	7.1% (0.2–33.9)	0.0% (0.0–23.2)	0.0% (0.0–23.2)	**0.368**	57.1% (28.9–82.3)	57.1% (28.9–82.3)	35.7% (12.8–64.9)	35.7% (12.8–64.9)	**0.212**
MES 3	54	0	0.0% (0.0–6.6)	0.0% (0.0–6.6)	0.0% (0.0–6.6)	**NE**	59.3% (45.0–72.4)	83.3% (70.7–92.1)	38.9% (25.9–53.1)	38.9% (25.9–53.1)	**<0.001**

Values are percentages with exact 95% confidence intervals (Clopper–Pearson). P values are global Cochran's Q tests comparing WLI, LCI and CAD-EYE for detection and the four characterization methods for characterization. NE = not estimable because all methods had identical results.

Supplementary Table S2. Lesion detection and characterization performance according to lesion size.

Subgroup	n	Neoplastic	WLI miss (95% CI)	LCI miss (95% CI)	CAD-EYE miss (95% CI)	*P* Detection	CAD-EYE Accuracy	KUDO-IBD Accuracy	KUDO Accuracy	NICE Accuracy	*P* Characterization
<10 mm	240	18	6.7% (3.9–10.6)	2.9% (1.2–5.9)	0.0% (0.0–1.5)	**<0.001**	75.4% (69.5–80.7)	86.2% (81.2–90.3)	78.3% (72.6–83.4)	68.8% (62.5–74.6)	**<0.001**
≥10 mm	41	4	0.0% (0.0–8.8)	0.0% (0.0–8.8)	0.0% (0.0–8.8)	**NE**	65.0% (48.3–79.4)	85.0% (70.2–94.3)	47.5% (31.5–63.9)	37.5% (22.7–54.2)	**<0.001**

Values are percentages with exact 95% confidence intervals (Clopper–Pearson). P values are global Cochran's Q tests comparing WLI, LCI and CAD-EYE for detection and the four characterization methods for characterization. NE = not estimable because all methods had identical results.

Supplementary Table S3. Lesion detection and characterization performance according to anatomical location.

Subgroup	n	Neoplastic	WLI miss (95% CI)	LCI miss (95 % CI)	CAD-EYE miss (95% CI)	*P* Detection	CAD-EYE Accuracy	KUDO-IBD Accuracy	KUDO Accuracy	NICE Accuracy	*P* Characterization
Left colon	92	16	13.0% (6.9–21.7)	5.4% (1.8–12.2)	0.0% (0.0–3.9)	**<0.001**	81.5% (72.1–88.9)	89.1% (80.9–94.7)	87.0% (78.3–93.1)	79.3% (69.6–87.1)	**0.060**
Right colon	189	6	2.1% (0.6–5.3)	1.1% (0.1–3.8)	0.0% (0.0–1.9)	**0.050**	70.4% (63.3–76.8)	84.1% (78.1–89.0)	67.7% (60.6–74.3)	57.1% (49.8–64.3)	**<0.001**

Values are percentages with exact 95% confidence intervals (Clopper–Pearson). P values are global Cochran's Q tests comparing WLI, LCI and CAD-EYE for detection and the four characterization methods for characterization. NE = not estimable because all methods had identical results.

Supplementary Table S4. Lesion detection and characterization performance according to Paris morphology.

Subgroup	n	Neoplastic	WLI miss (95% CI)	LCI miss (95 % CI)	CAD-EYE miss (95% CI)	*P* Detection	CAD-EYE Accuracy	KUDO-IBD Accuracy	KUDO Accuracy	NICE Accuracy	*P* Characterization
Polypoid	226	7	2.7% (1.0–5.7)	0.9% (0.1–3.2)	0.0% (0.0–1.6)	**0.009**	77.4% (71.4–82.7)	86.3% (81.1–90.5)	73.5% (67.2–79.1)	61.9% (55.3–68.3)	**<0.001**
Non-polypoid	55	15	18.2% (9.1–30.9)	9.1% (3.0–20.0)	0.0% (0.0–6.5)	**<0.001**	60.0% (45.9–73.0)	83.6% (71.2–92.2)	76.4% (63.0–86.8)	74.5% (61.0–85.3)	**0.002**

Values are percentages with exact 95% confidence intervals (Clopper–Pearson). P values are global Cochran's Q tests comparing WLI, LCI and CAD-EYE for detection and the four characterization methods for characterization. NE = not estimable because all methods had identical results.
